# A Practical Gut‐On‐Chip Workflow for Imaging‐Based Assessment of Epithelial Barrier Dynamics During *E. coli* Co‐Culture

**DOI:** 10.1002/bit.70240

**Published:** 2026-06-17

**Authors:** Shih‐Wei Chiang, Chuan‐Yi Yang, Guan‐Yu Chen

**Affiliations:** ^1^ Division of Colorectal Surgery, Department of Surgery Taichung Veterans General Hospital Taichung Taiwan; ^2^ Department of Electrical and Computer Engineering National Yang Ming Chiao Tung University Hsinchu Taiwan; ^3^ Institute of Intelligent Bioelectrical Engineering, College of Electrical and Computer Engineering National Yang Ming Chiao Tung University Hsinchu Taiwan; ^4^ Anivance AI Corporation Zhubei City Hsinchu County Taiwan; ^5^ Department of Biological Science and Technology, College of Biological Science and Technology National Yang Ming Chiao Tung University Hsinchu Taiwan; ^6^ Center for Intelligent Drug Systems and Smart Bio‐devices (IDS2B) National Yang Ming Chiao Tung University Hsinchu Taiwan

**Keywords:** Caco‐2, *E. coli*, epithelial barrier, gut‐on‐chip, host–microbe interaction, microfluidics, mucus, perfusion, shear stress, tight junction

## Abstract

The intestinal epithelium and its resident microbiota form a dynamic interface that is central to gut homeostasis, but this interface is difficult to model with conventional tools. Static Transwell cultures lack physiological fluid shear, whereas animal models are costly and poorly suited to quantitative, high‐content imaging. Organ‐on‐a‐chip technology offers a way to recreate key physical and biological features of the gut, yet many existing gut‐on‐chip systems remain technically demanding or poorly standardized for routine use. Here, we developed and validated a practical gut‐on‐chip workflow that enables reproducible, imaging‐based quantification of epithelial barrier dynamics during short‐term co‐culture with *Escherichia coli*. Using a commercial two‐channel microfluidic chip and Caco‐2 cells, we first established a perfused intestinal model under low‐flow conditions (0.5–1 µL/min) and confirmed long‐term stability by phase‐contrast imaging, LIVE/DEAD staining, and immunofluorescence for ZO‐1, F‐actin, and wheat germ agglutinin (WGA). This protocol generated a confluent, polarized monolayer with continuous tight junctions and an apical glycoprotein mucus layer. We then introduced HADA‐labeled *E. coli* and systematically varied the perfusion rate during co‐culture. At 0.5 µL/min, the epithelial layer, although stable in monoculture, underwent rapid thinning and focal detachment within 4 days of co‐culture, indicating that low shear is insufficient to buffer bacterial overgrowth and metabolite accumulation. In contrast, Caco‐2 monolayers tolerated perfusion up to 10 µL/min without structural damage, and at this higher flow rate we achieved a stable, 24‐h analytical window in which epithelial integrity and bacterial distribution could be imaged reliably. Structural disruption only became evident when co‐culture was extended to four and 7 days. Taken together, these results identify perfusion rate as the decisive parameter for stabilizing host–microbe co‐culture on a gut‐on‐chip platform. The workflow described here couples a functionally validated epithelial barrier with an experimentally simple, yet robust, perfusion regime. It provides an accessible test‐bed for quantitative studies of early host–microbe interactions and can be readily extended with orthogonal barrier readouts, such as TEER or tracer permeability, in future work.

## 1 Introduction

The intestinal epithelium forms a single‐cell‐thick barrier that must reconcile two opposing tasks: allowing efficient absorption of nutrients and water while preventing bacterial translocation and uncontrolled inflammation. This balance is governed by the interplay between epithelial cells, their apical mucus layer, and intercellular tight junctions, all of which are dynamically regulated by the gut microbiota and local immune signals. Disruption of this finely tuned system—often labeled “barrier dysfunction”—is now recognized as a core feature of inflammatory bowel disease (IBD), colorectal cancer (CRC), and a range of metabolic and systemic disorders (De Gregorio et al. 2022; Marrero et al. 2021; Gou et al. 2021; Quaglio et al. 2022).

Despite this central role, it remains difficult to study barrier dynamics under conditions that realistically reflect the physical and biochemical microenvironment of the gut. Conventional in vitro models rely heavily on Caco‐2 monolayers grown on static Transwell inserts. These platforms are inexpensive and straightforward to use, but they lack physiological shear stress and luminal flow, which are crucial for epithelial polarization, mucus turnover, and spatial control of microbial colonization (Afzaal et al. 2022; Qiu et al. 2022; Taavitsainen et al. 2024). Such static systems are also prone to artifacts during co‐culture with bacteria: local depletion of nutrients, accumulation of toxic metabolites, and runaway bacterial overgrowth can rapidly collapse the epithelium and obscure the biology of interest.

Animal models, on the other hand, offer systemic complexity but come with their own limitations: inter‐species differences, poor control over microbiota composition, ethical constraints, and limited suitability for high‐content imaging or mechanistic dissection at cellular resolution (Di Vincenzo et al. 2024; Xian et al. 2023; Valiei et al. 2023). These constraints have created a translational gap between basic microbiome research and clinically relevant applications, such as precision therapeutics for IBD and CRC.

Organ‐on‐a‐chip (OoC) technology has emerged as a powerful bridge across this gap. In these microfluidic platforms, cells are cultured under controlled perfusion and, in some cases, mechanical stretch, closely mimicking the physical conditions that tissues experience in vivo (Poceviciute and Ismagilov 2019; Jalili‐Firoozinezhad et al. 2019; Carvalho et al. 2023; Wheeler et al. 2024; Strelez et al. 2021). Lung‐on‐a‐chip systems, for example, have elegantly demonstrated how cyclic strain and air–liquid interfaces modulate barrier behavior and responses to pathogens or nanoparticles (Beaurivage et al. 2019; Li et al. 2022). Building on this conceptual advance, a series of gut‐on‐chip devices have been developed to recreate villus formation, mucus secretion, and microbial colonization under dynamic flow (Donkers et al. 2021; Lin et al. 2022a; Yang et al. 2022a). These systems have revealed new aspects of host–microbe crosstalk, including the influence of shear stress on Caco‐2 differentiation and the conditions required to sustain complex anaerobic microbiota consortia. Jalili‐Firoozinezhad et al. 2019; Carvalho et al. 2023; Chen et al. 2022.

Yet, despite this rapid progress, there is still a practical barrier to broader adoption of gut‐on‐chip models in laboratories that are primarily clinically oriented. Many reported platforms involve bespoke device fabrication, custom vacuum‐driven peristalsis mechanisms, or complex anaerobic infrastructure, which can be technically demanding and time‐consuming to implement. Furthermore, some studies focus on sophisticated biological endpoints but provide only limited procedural detail on device conditioning, perfusion setup, or co‐culture stability, making it difficult for other groups to reproduce the work or adapt it to their own questions (Yang et al. 2022b; Lin et al. 2022b).

Critically, while it is widely acknowledged that fluid shear stress promotes intestinal barrier function, the specific relationship between perfusion conditions and the stability of host–microbe co‐culture remains incompletely defined. Reviews and numerical studies have proposed shear stress ranges to support Caco‐2 polarization, and several groups have highlighted dynamic flow as a key parameter for physiologically relevant modeling (Kim et al. 2012; Kim and Ingber 2013; Fois et al. 2021). However, relatively few experimental studies have systematically interrogated how flow rate alone—without additional mechanical stretch—dictates whether an epithelial monolayer survives or collapses in the presence of bacteria over experimentally useful time frames.

In the context of colorectal surgery and gastroenterology, a methodologically simple yet quantitatively robust platform would be especially valuable. Surgeons and clinicians increasingly encounter patients whose disease courses are shaped by microbiota‐driven inflammation, but they often lack experimentally tractable models to test how patient‐derived microbes or candidate therapeutics interact with the intestinal barrier. A gut‐on‐chip platform that uses commercially available devices, standard cell lines, and straightforward perfusion control—if carefully validated—could serve as an entry point for such translational studies.

With this motivation, we set out to develop a practically deployable gut‐on‐chip workflow that emphasizes: (i) reproducible establishment of a functionally mature epithelial barrier; (ii) deliberate optimization of perfusion conditions to stabilize bacterial co‐culture; and (iii) compatibility with high‐content imaging as the primary quantitative readout. Using Caco‐2 cells cultured in a two‐channel microfluidic device, we first established a long‐term stable monoculture under low‐flow conditions and confirmed barrier maturity via ZO‐1 and F‐actin staining and WGA‐detected mucus. We then introduced fluorescently labeled *Escherichia coli* and systematically varied the perfusion rate, asking a simple but crucial question: at what flow rate does the epithelium remain structurally intact long enough to capture the early dynamics of host–microbe interaction?

Our findings reveal that while low‐flow culture (0.5–1 µL/min) is sufficient to maintain a mature Caco‐2 monolayer in isolation, the same conditions fail once bacteria are introduced, leading to rapid epithelial degradation. Conversely, a higher flow rate of 10 µL/min, corresponding to physiologically relevant low shear stress, (Kim et al. 2012; Bein et al. 2018), preserves barrier integrity for at least 24 h during *E. coli* co‐culture. This 24‐h window provides a robust analytical timeframe for imaging‐based quantification of bacterial adhesion, early barrier disruption, and subsequent recovery or failure.

In the following sections, we describe this workflow in detail and discuss how it can serve as a foundation for more complex, patient‐centered gut‐on‐chip studies.

## 2 Materials and Methods

This section details the experimental procedures used to establish the gut‐on‐chip model, validate epithelial barrier function, and optimize perfusion conditions for co‐culture with *E. coli*. Unless otherwise stated, all reagents were used as supplied by the manufacturer.

### 2.1 Cell Culture

Caco‐2 cells (ATCC HTB‐37) were maintained in Minimum Essential Medium (MEM; Corning 10‐009‐CV) supplemented with 20% fetal bovine serum (FBS; Corning) and 1% Antibiotic‐Antimycotic solution (Thermo Fisher Scientific SV30010). Cultures were incubated at 37°C in a humidified atmosphere of 5% CO₂. Cells were subcultured at 70%–80% confluence using 0.25% trypsin‐EDTA (Cytiva) and replated at a 1:4 split ratio in T75 flasks. To minimize passage‐related variability, only cells between passages 20 and 23 were used for chip experiments, consistent with common practice in gut‐on‐chip studies (Yang et al. 2022a; Kim et al. 2012).

### 2.2 Bacterial Culture and Fluorescent Labeling

*Escherichia coli* (BCRC 14902) was maintained on Tryptic Soy Agar (TSA) plates at 37°C. For co‐culture, a single colony was inoculated into Tryptic Soy Broth (TSB) supplemented with 0.8 mM 7‐Hydroxycoumarin‐Amino‐d‐Alanine (HADA; MedChemExpress HY‐131045) and incubated overnight at 37°C with shaking at 200 rpm. HADA incorporation provided a stable fluorescent signal suitable for microscopy‐based tracking of bacteria. The overnight culture was harvested by centrifugation (5000*g*, 3 min), washed twice in basal MEM to remove residual dye, and resuspended in antibiotic‐free complete MEM at 1 × 10⁷ CFU/mL for seeding into the microfluidic chip.

### 2.3 Gut‐On‐Chip Device Preparation and Coating

Experiments were conducted using a commercial microphysiological system and two‐channel organ‐on‐chip platform (Anivance AI Corporation, Hsinchu, Taiwan) consisting of parallel microchannels separated by a porous membrane. Devices were sterilized by flushing both channels with 70% ethanol, followed by UV exposure for 30 min in a biosafety cabinet.

To promote epithelial attachment, the apical channel was coated with 0.3 mg/mL type I collagen (Thermo Fisher Scientific A1048301) in 0.02 M acetic acid. The coating solution was introduced into the apical channel and incubated overnight at 4°C. Prior to cell seeding, residual collagen solution was removed and the channels were rinsed gently with MEM to equilibrate the surface.

### 2.4 Establishment of the Perfused Epithelial Model

Caco‐2 cells were resuspended at 1–3 × 10⁶ cells/mL in complete MEM and introduced into the apical channel using gentle pipette loading. Chips were placed in a humidified incubator at 37°C and 5% CO₂ and kept under static conditions overnight to allow initial adhesion.

The following day, continuous perfusion was initiated in both apical and basal channels using a peristaltic pump. Unless otherwise specified, low‐flow culture was performed at 0.5 or 1 µL/min, while high‐flow experiments used 2 or 10 µL/min. Fresh medium reservoirs were replaced every 2–3 days. Epithelial cultures were maintained under perfusion for up to 14 days to allow barrier maturation before co‐culture with bacteria.

### 2.5 Gut–Microbiota Co‐Culture Protocol

For co‐culture experiments, chips were first switched to antibiotic‐free complete MEM for at least 24 h to avoid carry‐over of antimicrobials. HADA‐labeled *E. coli* were introduced into the apical channel at 1 × 10⁷ CFU/mL under static conditions and allowed to adhere for 3 h at 37°C. After this attachment phase, perfusion was resumed in both channels at the designated flow rate (0.5, 2, or 10 µL/min). Co‐cultures were monitored for up to 7 days, with imaging performed at defined time points (Day 0, 1, 4, and 7).

### 2.6 Viability Staining

Cell viability within the epithelial monolayer was evaluated using the LIVE/DEAD Cell Imaging Kit (Invitrogen R37601). Both channels were gently rinsed with DPBS to remove serum proteins, followed by introduction of the Live Green/Dead Red dye solution according to the manufacturer's protocol. After a 15‐min incubation at room temperature, chips were imaged using a fluorescence microscope equipped with FITC (live cells, green) and Texas Red (dead cells, red) filter sets. This assay was performed at representative time points during long‐term monoculture (Day 7 and Day 14) and, selectively, during co‐culture.

### 2.7 Immunofluorescence Staining and Confocal Microscopy

To characterize barrier maturity, chips were fixed and stained for tight junctions, cytoskeletal organization, and mucus production. For ZO‐1 and F‐actin, cells were fixed with BD fixation buffer (BD Biosciences 554655) for 20 min, washed, and blocked in 5% bovine serum albumin (BSA) in DPBS. Samples were incubated overnight at 4°C with anti‐ZO‐1 primary antibody (Invitrogen 33‐9100; 1:200), followed by Alexa Fluor 647‐conjugated secondary antibody (Jackson ImmunoResearch 115‐605‐003; 1:400) and phalloidin‐Alexa Fluor 568 (Invitrogen A12380) for F‐actin.

For mucus analysis, a separate set of chips was fixed with Carnoy's solution (60% ethanol, 30% chloroform, 10% glacial acetic acid) for 20 min and stained with wheat germ agglutinin (WGA)‐Alexa Fluor 555 (Invitrogen W32464). In all staining protocols, nuclei were counterstained with DAPI (Vector Laboratories H‐1200‐10), and chips were mounted in antifade medium prior to imaging.

Images were acquired on a Leica TCS SP8 confocal microscope (Leica Microsystems) using sequential scanning to minimize spectral overlap. Z‐stack images were collected to visualize three‐dimensional features such as brush‐border organization and mucus coverage.

### 2.8 Image Analysis and Qualitative Scoring

Phase‐contrast and fluorescence images were imported into FIJI/ImageJ for basic processing. For descriptive analysis, epithelial monolayers were scored qualitatively for confluence, uniformity, and structural integrity (intact, locally thinned, or detached). Bacterial signal intensity and distribution were evaluated in the HADA channel to confirm successful seeding and persistence during co‐culture. While the present study focused on establishing a robust qualitative framework, the workflow is compatible with future quantitative segmentation and intensity‐based metrics.

### 2.9 Ethical and Biosafety Considerations

All procedures complied with the institutional biosafety regulations of Taichung Veterans General Hospital. The human research protocol entitled “Research on the Impact of Microbiota on Colonic Health Using a Colon Organ‐on‐a‐Chip Model” was approved by the Institutional Review Board (IRB) of Taichung Veterans General Hospital (IRB No. CE25084C, approval period 21 April 2025–20 April 2026).

## 3 Results

Our experiments were designed in two phases. First, we established and functionally validated a perfused Caco‐2 monolayer under low‐flow conditions. Second, we used this validated epithelial model to determine how perfusion rate influences the stability of co‐culture with *E. coli*.

### 3.1 Stable Long‐Term Epithelial Monoculture Under Low‐Flow Perfusion

**Figure 1:**
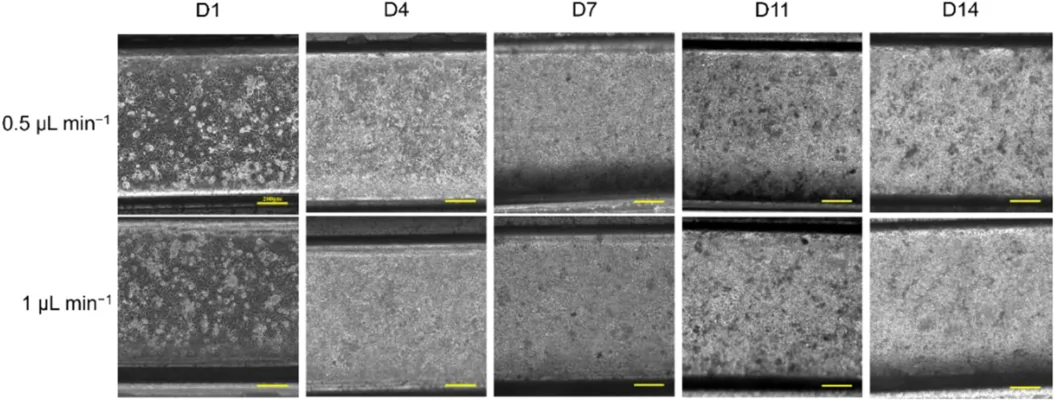
Dynamic bright‐field images of Caco‐2 cells cultured in a colon‐on‐a‐chip under different flow rates. Caco‐2 cells were dynamically perfused at 0.5 or 1 μL/min and monitored over time (D1–D14). Cells maintained stable attachment and continuous growth up to day 14 under both conditions.

Caco‐2 cells seeded into the collagen‐coated apical channel attached efficiently and spread to form a contiguous monolayer within several days. Under continuous perfusion at 0.5 or 1 µL/min, phase‐contrast imaging showed that the monolayer remained confluent and morphologically stable through at least Day 14 (Figure 1). No gross defects, gaps, or detachment were observed at either flow rate, indicating that these conditions are sufficient to support long‐term monoculture in the microfluidic device.

**Figure 2:**
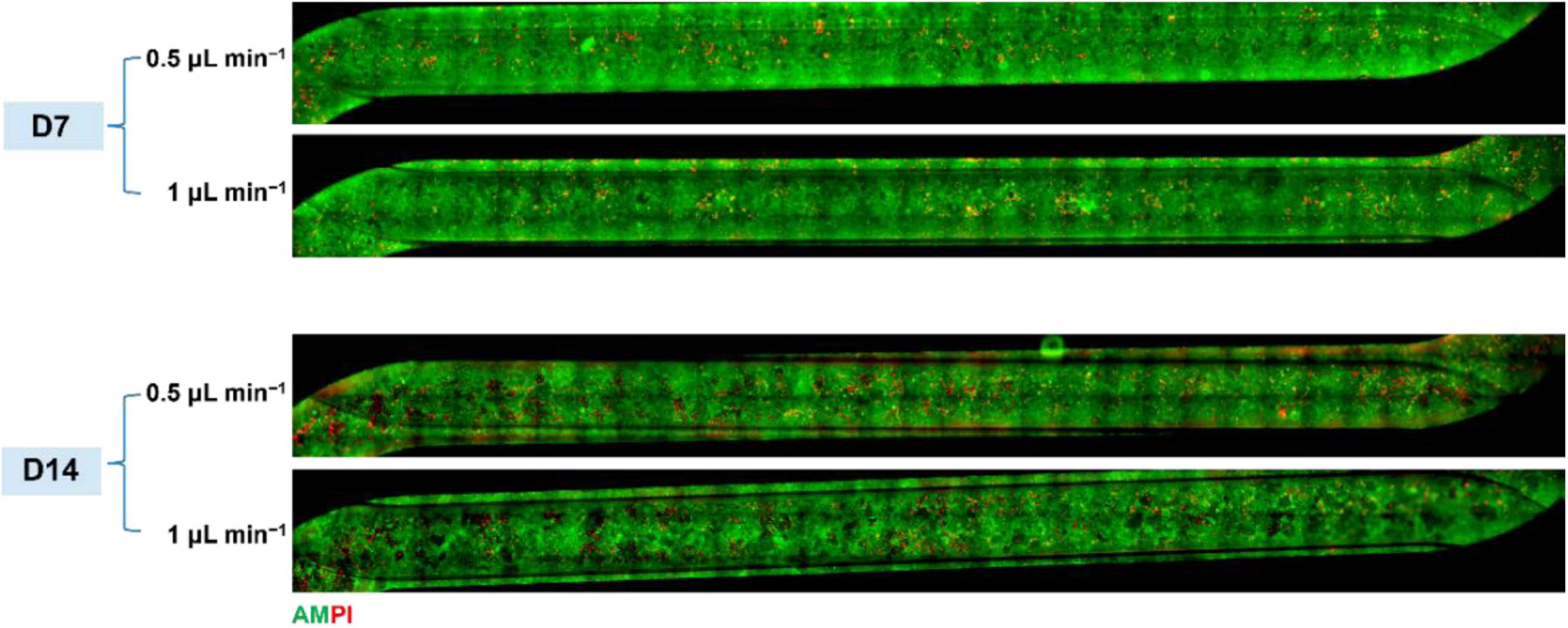
Live/dead viability staining of Caco‐2 cells in the chip during dynamic culture. Representative AM/PI staining shows live cells (AM, green) and dead cells (PI, red), demonstrating that most cells remained viable under both flow conditions, supporting the stability of the culture parameters.

LIVE/DEAD staining at Days 7 and 14 confirmed high levels of viability under both low‐flow conditions (Figure 2). The fluorescent images were dominated by green signal (live cells), with only sporadic red signal (dead cells). This result suggests that neither shear stress nor medium renewal at 0.5–1 µL/min imposes significant stress on the epithelial monolayer in the absence of bacteria.

### 3.2 The Perfused Monolayer Exhibits Tight Junctions, Polarization, and an Apical Mucus Layer

**Figure 3:**
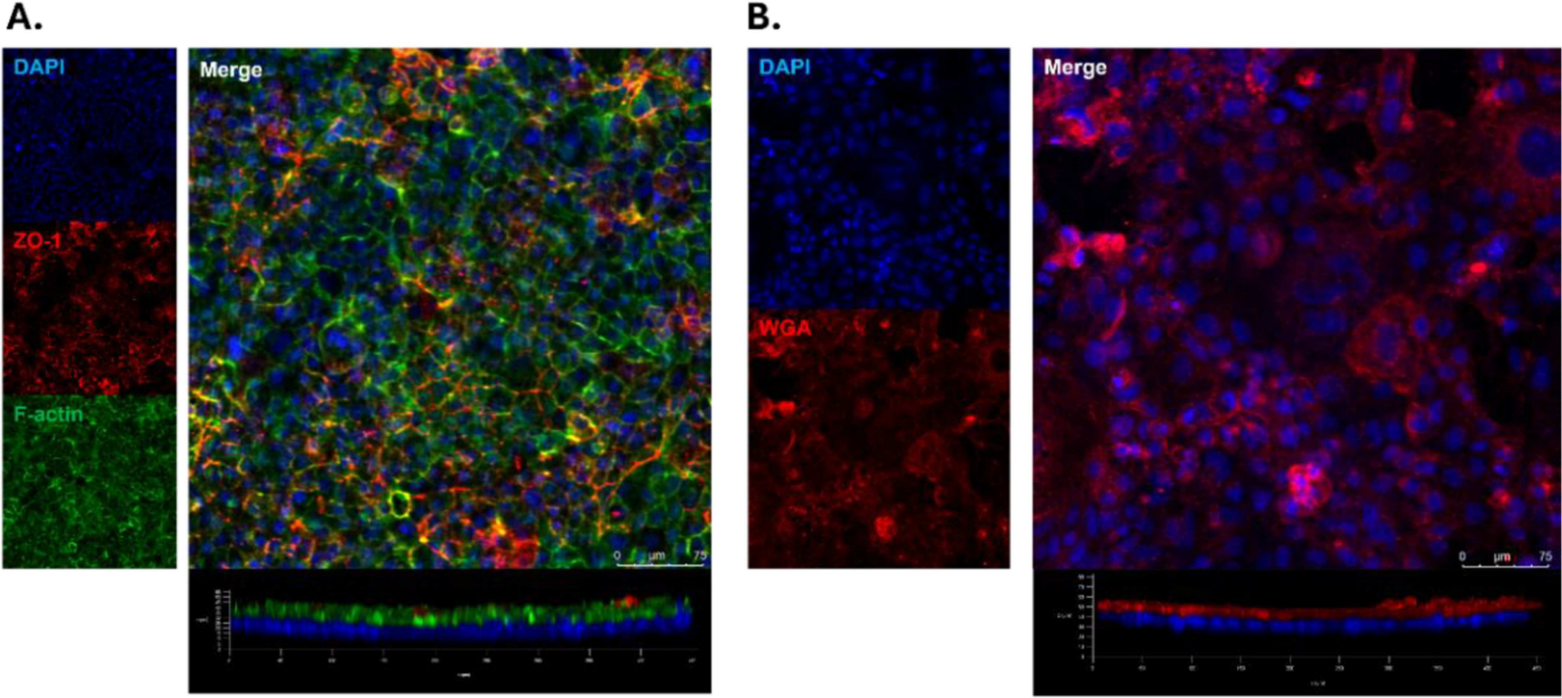
Immunofluorescence characterization of functional epithelial features in chip‐cultured Caco‐2 cells. (A) ZO‐1 (red) shows continuous junctional localization at cell borders, indicating intact tight junctions; F‐actin (green) displays apical brush‐like organization consistent with epithelial polarization; nuclei were counterstained with DAPI (blue). Merged images and Z‐stack side views demonstrate a three‐dimensional epithelial layer. (B) WGA (red) uniformly covers the apical surface, indicating formation of a stable glycoprotein/mucus layer; DAPI (blue) marks nuclei. Z‐stack side views confirm apical enrichment of the mucus layer.

We next evaluated whether the low‐flow culture conditions produced a functionally mature barrier. Confocal imaging of ZO‐1 revealed a continuous ring‐like distribution at cell–cell boundaries, with minimal interruptions, consistent with well‐formed tight junctions (Figure 3A). F‐actin staining showed a dense, brush‐border‐like structure at the apical surface and organized cortical actin at the lateral membrane, indicating proper polarization of Caco‐2 cells in the microfluidic environment.

**Figure 4:**
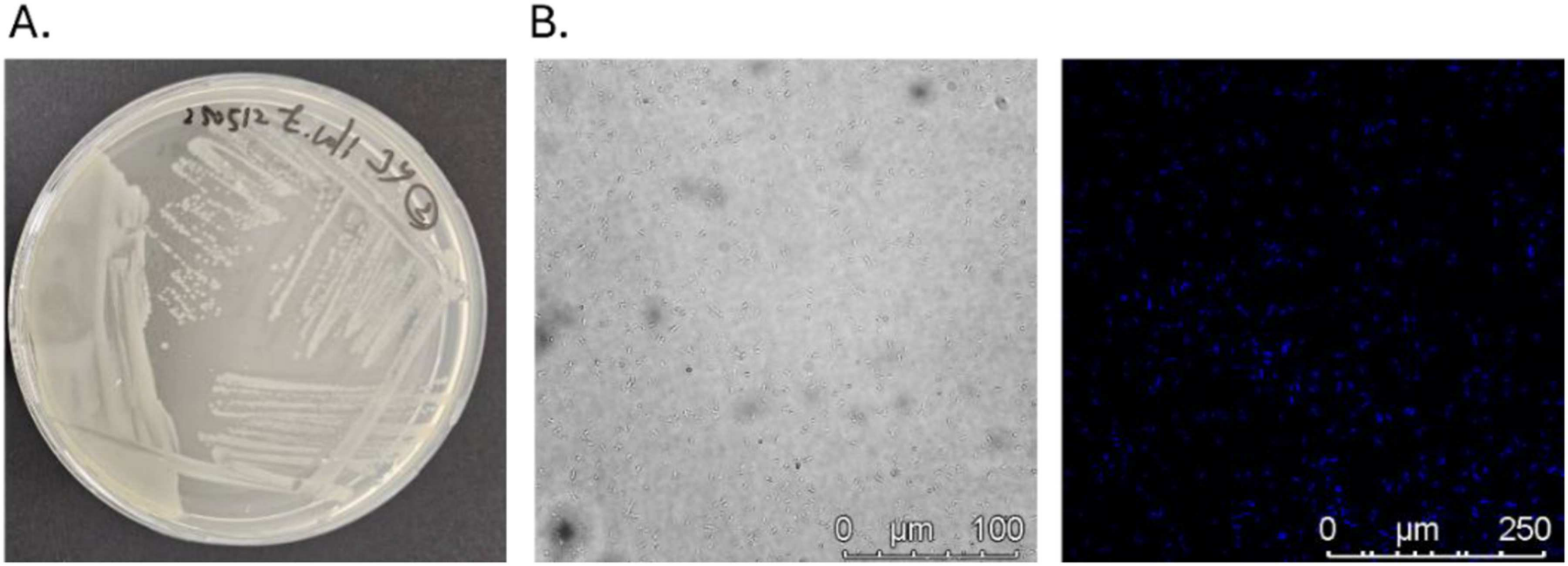
Establishment of Escherichia coli culture and fluorescence labeling workflow. (A) Representative colony growth of *Escherichia coli* on TSA plates. (B) HADA fluorescence labeling shows stable bacterial fluorescent signals, supporting its use for subsequent tracking of bacterial distribution in the chip system.

Mucus production was assessed using WGA staining. A uniform WGA‐positive layer covered the apical surface of the monolayer (Figure 3B), and Z‐stack reconstruction localized this glycoprotein‐rich signal above the nuclei, confirming that an extracellular mucus layer had formed. Together, these imaging results establish that our perfused Caco‐2 model reproduces not only the structural organization but also key functional features of an intestinal epithelial barrier, providing a robust starting point Figure 4. for host–microbe interaction studies.

### 3.3 Low‐Flow Perfusion Is Insufficient to Maintain Epithelial Integrity During *E. coli* Co‐Culture

To test the impact of perfusion rate on co‐culture stability, we first introduced HADA‐labeled *E. coli* into the established epithelial model under low‐flow conditions (0.5 µL/min). As expected, bacteria were readily detectable within the apical channel immediately after seeding (Day 0) and at 24 h (Day 1), confirming successful colonization.

**Figure 5:**
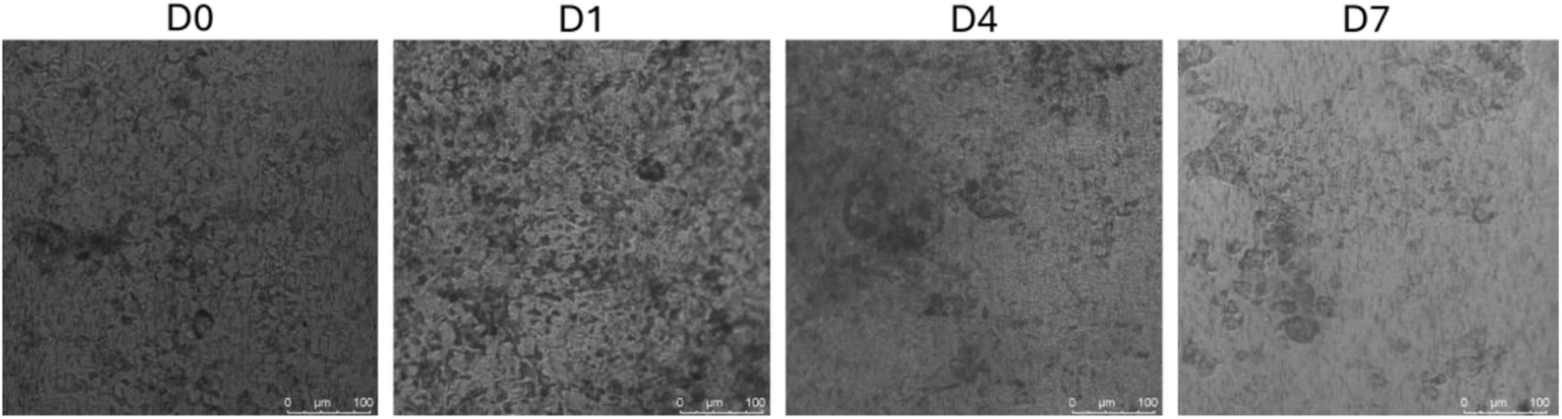
Co‐culture of Caco‐2 cells and E. coli under low‐flow conditions. Representative bright‐field images of the epithelial layer during co‐culture at 0.5 μL/min. The epithelium appeared intact at D1, but progressively thinned with focal detachment from D4 onward, indicating that low‐flow perfusion was not favorable for maintaining stable host–microbe co‐culture.

However, structural changes in the epithelial layer emerged rapidly thereafter. By Day 4, phase‐contrast images revealed localized thinning of the monolayer and areas of partial detachment from the membrane (Figure 5). These defects progressed by Day 7, with larger regions showing compromised integrity. Importantly, this degradation occurred under conditions that were perfectly adequate for long‐term monoculture, implicating the presence of bacteria—rather than flow per se—as the trigger for epithelial collapse under low‐flow conditions.

This observation is consistent with the notion that insufficient perfusion allows local accumulation of bacterial biomass and metabolites, leading to steep gradients in oxygen, pH, and nutrient availability. Under such conditions, epithelial cell death and detachment may reflect environmental collapse rather than physiologically meaningful responses to controlled microbial stimuli.

### 3.4 Caco‐2 Monolayers Tolerate Higher Perfusion Rates Without Loss of Viability

**Figure 6:**
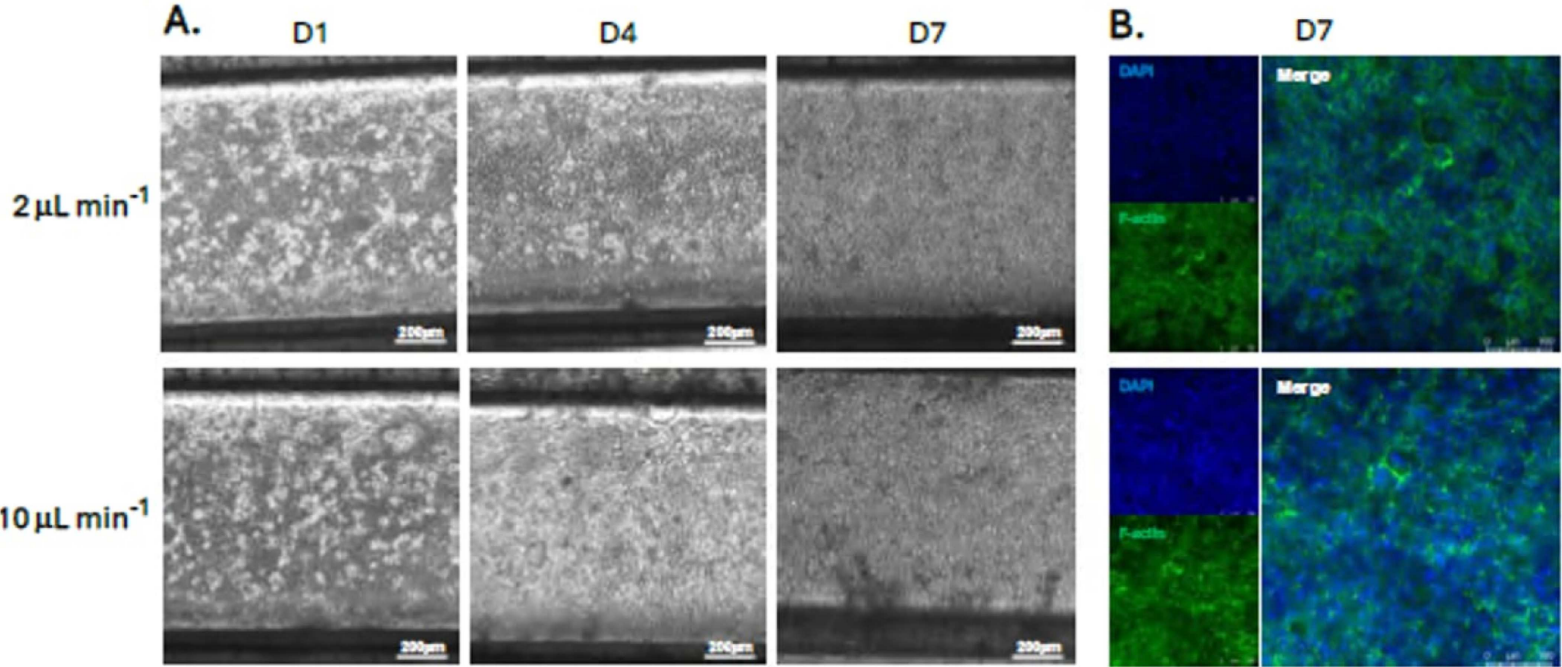
Optimization of dynamic culture conditions for maintaining Caco‐2 epithelial integrity. (A) Bright‐field images of Caco‐2 cells cultured at 2 or 10 μL/min over time (D1–D7). Higher flow (10 μL/min) better preserved epithelial organization and structural integrity compared with 2 μL/min. (B) Immunofluorescence at D7 showing F‐actin (green) cytoskeletal organization and DAPI (blue) nuclear staining; the epithelial layer exhibits tighter packing and improved structural maintenance under higher flow.

To determine whether increasing the perfusion rate would damage the epithelium before co‐culture, we next cultured Caco‐2 cells in monoculture at higher flow rates of 2 and 10 µL/min. Under both conditions, cells attached uniformly and formed confluent monolayers by Day 1. Over the following week, phase‐contrast imaging showed that the epithelial layer remained intact and that cell morphology became more compact and homogeneous, particularly at 10 µL/min (Figure 6A).

Immunofluorescence staining on Day 7 confirmed that F‐actin organization and nuclear morphology were well preserved at the highest tested flow rate (Figure 6B). These findings indicate that Caco‐2 cells can tolerate, and may even benefit from, modestly increased shear stress, in line with previous reports that low‐to‐moderate shear promotes barrier function and differentiation in gut‐on‐chip models (Kim et al. 2012; Fois et al. 2021).

### 3.5 High‐Flow Perfusion Enables a Stable 24‐h Analytical Window for Host–Microbe Interaction

**Figure 7:**
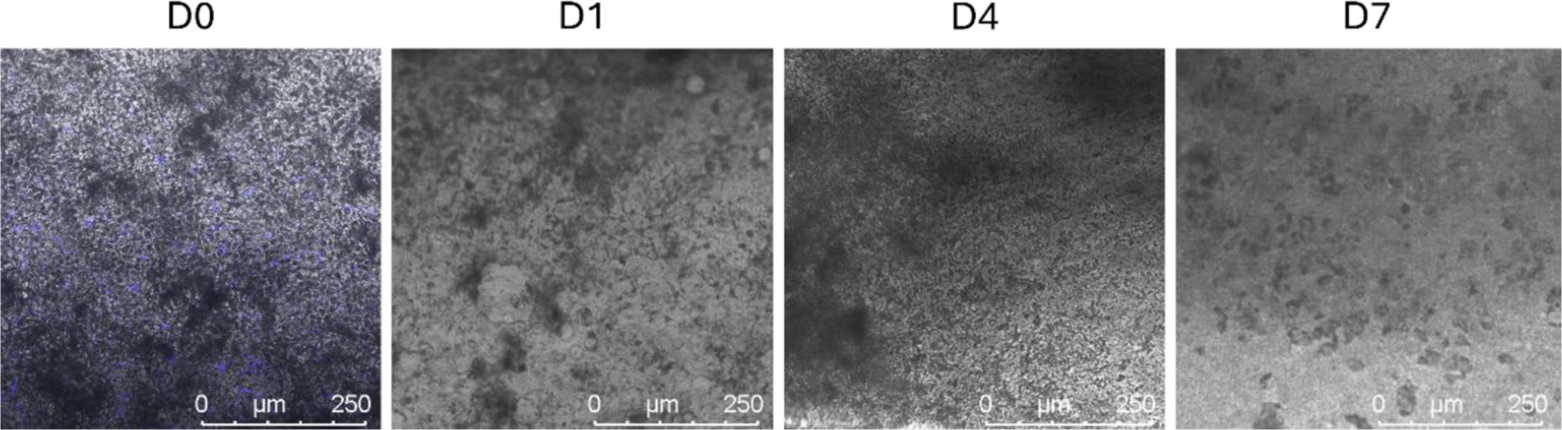
Co‐culture of Caco‐2 cells and *Escherichiacoli* under optimized high‐flow conditions. At 10 μL/min, HADA labeling enables clear visualization of bacteria at D0, confirming successful bacterial introduction. The epithelial layer remains intact at D1; however, bright‐field images at D4 and D7 show onset of epithelial structural damage, suggesting that further optimization is required for long‐term stable co‐culture.

Having established the epithelial tolerance to higher flow, we performed *E. coli* co‐culture at 10 µL/min. HADA fluorescence confirmed efficient bacterial loading into the apical channel at Day 0, with bacteria distributed along the epithelial surface (Figure 7). Crucially, after 24 h of co‐culture (Day 1), the Caco‐2 monolayer remained structurally intact, with no obvious thinning or detachment. Cell morphology was comparable to monoculture controls, indicating that the combination of high perfusion and bacterial presence did not acutely damage the epithelium.

When the co‐culture was extended to Days 4 and 7, however, phase‐contrast imaging revealed progressive structural changes. The monolayer became less uniform, and cell density decreased in some regions, suggesting that long‐term exposure to actively replicating bacteria still challenges barrier integrity even under high‐flow conditions. These observations define practical operational limits for the current system: a robust, reproducible 24‐h window for quantitative imaging of early host–microbe interactions, followed by gradual deterioration that may be useful for studying barrier failure but less suitable for routine screening.

Collectively, these results demonstrate that perfusion rate—rather than device geometry or cell line choice alone is a key factor determining whether a gut‐on‐chip co‐culture experiment provides a stable platform or rapidly degenerates into a non‐physiological collapse.

## 4 Discussion

In this work, we set out to build a gut‐on‐chip workflow that is technically straightforward and experimentally reproducible for studying early host–microbe interactions under controlled perfusion conditions. Rather than aiming to establish new mechanistic insights, our goal was to define a practical and stable operating regime that enables reliable imaging‐based assessment of epithelial barrier dynamics during short‐term co‐culture. Using a commercially available two‐channel device, a widely used human intestinal cell line, and standard laboratory equipment, we established a perfused epithelial model that recapitulates essential features of the intestinal barrier and then used it to dissect the role of perfusion rate in co‐culture stability with *E.coli*.

### 4.1 Perfusion as the Critical Design Parameter

Our most striking finding is the sharp contrast in epithelial outcomes between low‐ and high‐flow co‐culture conditions. At 0.5 µL/min, Caco‐2 monolayers remained viable and structurally intact for at least 2 weeks in monoculture, yet the same conditions led to pronounced degradation within 4 days of co‐culture. At 10 µL/min, by contrast, the epithelium tolerated 24 h of co‐culture without apparent structural damage. Based on channel dimensions, this flow rate corresponds to an estimated wall shear stress in the range of approximately 10⁻³–10⁻² dyn/cm², consistent with previously reported reported range in gut‐on‐chip systems (Kim et al. 2012; Fois et al. 2021).

These observations align with and extend prior work emphasizing the importance of shear stress in gut‐on‐chip systems. Kim and colleagues, in their seminal “human gut‐on‐a‐chip” studies, showed that exposing Caco‐2 cells to flow and peristalsis‐like motions promoted villus formation, mucus secretion, and enhanced barrier function (Donkers et al. 2021; Lin et al. 2022a). Subsequent numerical and experimental analyses have suggested shear stress values on the order of 0.02–0.04 dyn/cm² as a useful range for intestinal models (Kim et al. 2012; Fois et al. 2021; Bein et al. 2018). Our data suggest that when bacteria are present, perfusion must not only meet the cells' mechanical needs but also provide sufficient convective transport to dilute metabolites, remove shed cells, and prevent localized bacterial overgrowth. Importantly, within the present experimental design, it is not possible to decouple the respective contributions of mechanical shear stress and convective mass transport. The observed stabilization at higher perfusion rates likely reflects a combination of these effects, including improved nutrient renewal, metabolite dilution, and control of bacterial biomass, rather than a purely mechanobiological response.

In other words, flow acts as a coupled parameter that influences both mechanical cues and the surrounding chemical microenvironment. Too little flow may leave the system vulnerable to non‐physiological collapse, whereas excessively high shear could damage the epithelium or strip away loosely adherent commensals. Our results identify 10 µL/min in this particular device as a useful compromise that stabilizes co‐culture for at least 24 h while remaining well tolerated by Caco‐2 cells.

### 4.2 A Practical Platform for Early‐Stage Host–Microbe Studies

By combining validated barrier maturation with high‐flow co‐culture, the present workflow yields a reproducible 24‐h analytical window for imaging‐based evaluation of epithelial structure and bacterial distribution. While these imaging‐based readouts provide structural insight into barrier integrity, they do not substitute for functional measurements such as TEER or paracellular permeability, which will be important to incorporate in future studies. This timeframe is particularly well suited for questions centered on bacterial adhesion, early tight‐junction remodeling, and rapid epithelial stress responses, which often unfold over a few hours to 1 day.

From a practical standpoint, this window aligns well with typical experimental schedules in hospital‐based laboratories: chips can be seeded and matured in advance, bacteria introduced on a chosen day, and imaging‐based readouts collected over the following 24 h without requiring continuous overnight supervision or elaborate automation. The reliance on imaging rather than specialized sensors also lowers the barrier to entry for laboratories that do not yet have integrated TEER or oxygen probes on chip. Nonetheless, our platform is compatible with emerging sensor‐integrated devices that provide real‐time barrier measurements and could be layered onto this workflow in future iterations (Kim and Ingber 2013).

### 4.3 Relation to Existing Gut‐On‐Chip Literature

Several reviews have cataloged the diverse landscape of gut‐on‐chip technologies, highlighting the variety of materials, channel architectures, and co‐culture strategies currently in use (Marrero et al. 2021; Xian et al. 2023; Bein et al. 2018; Valiei et al. 2023; Clarke et al. 2021). Some systems emphasize physiological realism, incorporating peristaltic deformation, co‐culture with endothelial cells, or complex anaerobic microbiota. Others focus on integration of biosensors for long‐term barrier monitoring or drug permeability assays (Carvalho et al. 2023; Kim and Ingber 2013; Wang et al. 2023).

Our contribution is deliberately more modest but complementary: we focus on defining a robust operating regime for a relatively simple, readily accessible platform. By doing so, we hope to make gut‐on‐chip technology more approachable to groups whose primary expertise lies in clinical gastroenterology or colorectal surgery rather than microfabrication. The explicit documentation of perfusion conditions, surface coating, cell passage range, and staining procedures is intended to facilitate direct replication or incremental adaptation, for example, by substituting patient‐derived organoids or incorporating immune cells (Kasendra et al. 2018). Accordingly, the present study should be interpreted as a workflow‐oriented contribution rather than a mechanistic investigation of barrier regulation.

### 4.4 Future Directions

While the current model successfully recapitulated key features of the intestinal barrier, several aspects could be further improved in future studies. First, we used a monoculture of Caco‐2 cells, which capture many features of intestinal epithelium but do not represent its full cellular diversity. Goblet cells, Paneth cells, enteroendocrine cells, and resident immune cells all contribute to barrier function and responses to microbes. Future iterations of this model could incorporate co‐cultures or stem‐cell‐derived organoids to better mimic this complexity (Kasendra et al. 2018; Li et al. 2022).

Second, we focused on a single commensal *E. coli* strain under aerobic conditions. While this choice allowed us to systematically explore perfusion effects without the added complexity of strict anaerobes, it does not fully represent the diverse microbial consortia found in the human colon. Sophisticated intestine‐on‐a‐chip platforms have shown that complex anaerobic microbiomes can be maintained under carefully controlled oxygen and flow conditions, (Jalili‐Firoozinezhad et al. 2019) but these setups require additional infrastructure. An intermediate step could be to test defined synthetic communities or clinical isolates associated with IBD or CRC, using our workflow as a baseline and gradually adapting flow and medium composition.

Third, our current analysis is primarily qualitative, relying on imaging‐based assessment of structural integrity rather than quantitative barrier metrics such as TEER, paracellular tracer flux, or single‐cell transcriptomics. The high‐content imaging data generated here—particularly the confocal characterization of ZO‐1, F‐actin, and mucus—nonetheless provide a mechanistic “ground truth” that can be correlated with future quantitative readouts. Integrating TEER electrodes or impedance sensors into the chip, as demonstrated in recent sensor‐integrated gut‐on‐chip designs, (Kim and Ingber 2013; Brandauer et al. 2025), would enable continuous, non‐invasive monitoring of barrier function alongside imaging. This represents a key limitation of the current study, particularly given that quantitative barrier metrics are widely used as standard readouts in organ‐on‐chip systems.

Finally, the long‐term co‐culture stability beyond 7 days remains to be optimized. Even at 10 µL/min, the epithelial monolayer began to deteriorate under sustained bacterial challenge. Strategies to prolong stability might include modulating inoculum density, using periodic media changes with bacteriostatic supplements, or applying peristalsis‐like mechanical deformation to enhance mucus turnover and detach excess bacteria, as shown in other gut‐on‐chip models (Donkers et al. 2021; Lin et al. 2022a; Kim et al. 2012).

### 4.5 Translational Implications

Despite these limitations, the present platform may serve as a starting point for future translational applications. For example, biopsies or resection specimens from patients with IBD or CRC could be used to derive organoids that are then seeded into the same chip architecture. Co‐culture with patient‐specific microbiota or defined therapeutic bacteria could potentially enable observation of barrier responses under controlled flow conditions, providing an ex vivo complement to clinical decision‐making (Kasendra et al. 2018; Li et al. 2022). Similarly, candidate drugs—including probiotics, small‐molecule anti‐inflammatories, or biologics—could be screened for their ability to preserve barrier integrity during bacterial challenge, helping to de‐risk investments before moving to costly animal or human studies.

More broadly, the workflow demonstrates how organ‐on‐a‐chip concepts can be translated from highly engineered prototypes into practical tools that fit within the constraints of hospital‐based laboratories. By focusing on a narrow but clinically relevant question—how perfusion rate controls epithelial stability during bacterial co‐culture—we provide a template that can be adapted and extended by others working at the interface of engineering, microbiology, and clinical medicine. We emphasize that the primary contribution of this study lies in defining an experimentally accessible and reproducible workflow, rather than establishing new biological mechanisms.

## 5 Conclusion

We have developed and validated a gut‐on‐chip workflow that combines a functionally mature Caco‐2 epithelial barrier with a systematically optimized perfusion regime for co‐culture with *E. coli*. Under low‐flow conditions, the epithelial monolayer is stable in monoculture but quickly deteriorates in the presence of bacteria. Increasing the perfusion rate to 10 µL/min, while well tolerated by the cells, yields a robust 24‐h analytical window in which barrier structure and bacterial distribution can be quantified by high‐content imaging.

These findings highlight perfusion rate as a key, and often under‐specified, parameter in gut‐on‐chip experiments, particularly when modeling host–microbe interactions. The practical protocol described here lowers the barrier for adoption of microfluidic intestinal models in clinically oriented laboratories and provides a platform that can be readily extended with more complex cell types, microbial communities, and quantitative readouts. In the long term, such accessible gut‐on‐chip systems may help bridge the gap between bench‐top experiments and personalized therapeutic strategies for patients with microbiota‐driven gastrointestinal disease.

## Author Contributions

S‐W.C. conceived and supervised the study, interpreted the data, and drafted the manuscript. C‐Y.Y. performed experiments. G‐Y.C. co‐designed the microfluidic experiments, optimized the chip protocols, and contributed to data interpretation and manuscript revision. All authors approved the final manuscript.

## Ethics Statement

All experimental procedures complied with institutional biosafety guidelines. The human research protocol “Research on the Impact of Microbiota on Colonic Health Using a Colon Organ‐on‐a‐Chip Model” was approved by the Institutional Review Board of Taichung Veterans General Hospital (IRB No. CE25084C; approval period 21 April 2025–20 April 2026).

## Conflicts of Interest

The authors declare no conflicts of interest.

## Data Availability

The data that support the findings of this study are available on request from the corresponding author. The data are not publicly available due to privacy or ethical restrictions.
